# Topological vulnerability explains fungal and bacterial stability differences in restoration of alpine grasslands

**DOI:** 10.3389/fmicb.2026.1767220

**Published:** 2026-02-23

**Authors:** Junguang Zhao, Qianqian Lu, Xiaoyu Zhang, Shikui Dong

**Affiliations:** 1 School of Science, Beijing Forestry University, Beijing, China; 2 School of Grassland Science, Beijing Forestry University, Beijing, China; 3 State Key Laboratory of Efficient Production of Forest Resources, Beijing Forestry University, Beijing, China

**Keywords:** community assembly, co-occurrence network, ecological restoration, keystone taxa, microbial succession

## Abstract

**Background:**

The alpine grasslands of the Qinghai-Tibetan Plateau (QTP) are critical ecosystems for regional climate regulation and biodiversity but are threatened by severe degradation. The success of ecological restoration in these fragile environments is intrinsically linked to the reassembly of belowground soil microbial communities. However, the specific successional trajectories and network-level mechanisms driving their reassembly during long-term restoration remain unclear.

**Objective:**

This study aimed to characterize the distinct successional trajectories of bacterial and fungal communities and to elucidate the topological mechanisms driving their transition along a long-term restoration chronosequence.

**Methods:**

Utilizing a space-for-time substitution approach on “Black Beach” degraded plots, we integrated consensus clustering with topological null model analyses to quantify community assembly processes. We evaluated the transition from stochastic to deterministic assembly by calculating Modularity Z-scores and analyzing structural changes in co-occurrence networks.

**Results:**

Microbial reassembly follows a predictable, non-linear trajectory comprising an early “Chaos Stage” (structural fragmentation) and a later “Recovery Stage” (high cohesion and deterministic interactions). Bacteria and fungi exhibited decoupled temporal dynamics: bacteria displayed an oscillatory “fast-in, fast-out” pattern, whereas fungi followed a “lagged but persistent” trajectory. Network analysis revealed a shift from random associations to competitive dominance. While the Recovery Stage exhibited increased complexity, it revealed a hidden topological fragility where stability became heavily dependent on specific keystone taxa, particularly within fungal networks.

**Conclusions:**

Microbial succession is driven by a regime shift from stochastic to deterministic network assembly. The identified topological fragility constitutes a core mechanism underlying the differential stability of fungi relative to bacteria. Monitoring network vulnerability and kingdom-specific decoupling provides a more accurate indicator for evaluating long-term ecosystem restoration success than species diversity alone.

## 1 Introduction

The alpine grasslands of the Qinghai-Tibetan Plateau (QTP) are the critical ecosystems for global climate regulation and biodiversity conservation. However, in recent decades, driving factors including climate change, overgrazing and anthropogenic intervention have led to severe degradation in these fragile ecosystems. This degradation threatens regional ecological security and may change global climate patterns through carbon cycle feedbacks (Dong et al., 2020). Consequently, ecological restoration has become an essential priority. Previous studies have confirmed that restoration success depends not only on aboveground vegetation but also on the re-establishment of the belowground microbial community (Du et al., 2025). Microorganisms, particularly bacteria and fungi, drive key ecosystem nutrient cycling and organic matter decomposition processes, serving as the subterranean engine that maintains grassland ecological function (Griffiths and Philippot, 2013).

Bacteria and fungi often exhibit significant differences in their responses to environmental filtering. For example, bacterial diversity is more readily regulated by soil pH and N:P ratio, whereas fungal diversity is more closely associated with mean annual temperature and aboveground net primary productivity (Liu et al., 2020). This divergence not only implies different assembly processes but also suggests they possess different stability characteristics during ecological succession (Jiao and Lu, 2020). Despite this, the understanding of their difference in stability and the complexity of their interaction networks during long-term restoration remains insufficient. The stability of the microbial community is a cornerstone of ecosystem function. It is governed not only by species composition but also by the complex interaction networks within the community, which range from cooperation to competition (De Vries et al., 2018). The Stress Gradient Hypothesis (SGH) provides a classic theoretical framework for such shifts in interaction patterns. This hypothesis was initially proposed to explain how biotic interactions in plant communities change across stress gradients, predicting that under high environmental stress, organisms tend to favor cooperation to enhance resource utilization and resist stress. Conversely, under low stress conditions competitive relationships become dominant (Maestre et al., 2009). Subsequent research has confirmed its applicability to microbial networks. For instance, Hernandez et al. (2021) found that the proportion of positive associations in microbial networks increased significantly under high-stress conditions, confirming that cooperation is a critical strategy for microbes to cope with extreme environments. Although SGH has been validated in various ecosystems, its application to the microbial network dynamics of alpine grassland restoration need to be further testified.

A recent foundational study Du et al. (2025), which used the same dataset as ours and provided critical background for this research, revealed two key findings at the phenomenal and mechanistic levels. Phenomenally, Du et al. (2025) observed that during the long-term restoration of alpine grasslands, bacterial communities exhibit higher compositional resistance and resilience than fungal communities, with the latter being more volatile and assembled via stochastic processes. Mechanistically, this discrepancy is closely related to core microbes' ability to maintain network resilience. Qiao et al. (2024) have corroborated this mechanistic link: they confirmed that core species enhance plant health by increasing network complexity and stability, providing direct evidence for the regulation of network resilience by core microbes as reported by Du et al. (2025). Despite these insights, a critical mechanistic question remains unresolved: Why is fungal community stability lower? This gap is addressed by paralleling findings from Jiao et al. (2022) in arid ecosystems, who showed that stochastically dominated fungal communities are more vulnerable to environmental fluctuations due to a lack of stable network support. This suggests that alpine grassland fungal networks may share a similar vulnerability, likely rooted in their network topology. For example, variations in the number, connectivity or modularity of keystone taxa could directly drive differences in disturbance tolerance between bacteria and fungi. Qiao et al. (2024) have further confirmed that the loss of core keystone nodes significantly reduces microbial network complexity and stability and taxonomic groups may differ fundamentally in their dependency on these key nodes. However, quantitative evidence for the vulnerability of fungal networks in the QTP's grasslands is currently lacking. It remains unknown whether the observed stability difference between bacteria and fungi stems from differential dependency on keystone taxa.

This study aims to provide a quantitative explanation for the stability of bacterial and fungial community based on network topology. First, to address the nonlinear characteristics of the restoration process also observed by Du et al. (2025), we employ a methodology combining (clustering analysis and machine learning) to identify and validate discrete state shifts in the restoration (i.e., Chaos Stage and Recovery Stage). Second, we deconstruct the network structures within these stages to validate the applicability of the SGH mechanism in different recovery phases, aligning with the logic used by Hernandez et al. (2021) to parse stress gradient effects via network interactions. Finally, as the core of this study, we use network robustness simulations to quantitatively compare the topological vulnerability of the bacterial and fungal networks in the late recovery stage. The work of Yuan et al. (2021), which confirmed that network topological parameters (nodes, connectivity) are positively correlated with robustness and that topology directly determines community tolerance to disturbance, provides a methodological foundation for our simulations. Our core hypotheses are: (1) Alpine grassland restoration is a nonlinear state shift process that can be classified into discrete and validatable ecological stages. (2) The mechanism of this transition is SGH-compliant, shifting from cooperation-dominated to competition-dominated network interactions. (3) The fungal instability is a direct consequence of its network topology that the fungal network is more fragile than the bacterial network and exhibits extreme dependence on the loss of its key nodes.

## 2 Materials and methods

### 2.1 Study site and experimental design

The study was conducted in Dawu town, Maqin country, a typical alpine area characterized by a mean annual temperature (MAT) of 0.28°C and an average annual rainfall of 516 mm. The experimental sites were established on comparable severely degraded alpine grasslands (classified as “Black Beach” type) where human-induced restoration practices of re-vegetation had been underway since 2000. To ensure a standardized initial baseline across all restoration ages, the sites were first plowed to remove poisonous plants and annual weeds, after which a mixture of four native grass species was seeded. The species included *Elymus nutans, Elymus sibiricus, Festuca sinensis*, and *Poa crymophila*. The respective seeding rates were 6kg·hm^−2^ for *E. nutans*, 6kg·hm^−2^ for *E. sibiricus*, 4.5kg·hm^−2^ for *F. sinensis* and 1.5kg·hm^−2^ for *P. crymophila*, according to a seeding density ratio of 1:1:1:1. Following reseeding, all sites were immediately fenced to prevent disturbance and allow for progressive succession, with no further management (e.g., fertilization or irrigation) applied.

### 2.2 Sample collection

Sampling was conducted in 2017 using a space-for-time substitution approach (chronosequence) to measure the soil microbe of restored grasslands at different years: 1 year (1 y), 4 years (4 y), 6 years (6 y), 8 years (8 y), 9 years (9 y), 12 years (12 y), 14 years (14 y), 16 years (16 y), and 18 years (18 y) (Walker et al., 2010). For comparison, non-degraded alpine grasslands and unrestored severely degraded alpine grasslands were also sampled as controls. For each treatment (each successional year and the controls), sampling was conducted across five spatially independent patches to serve as true biological replicates. Within each patch, a sampling quadrat was randomly placed, and three soil cores were randomly collected using an auger. These three cores were then mixed to create a single composite sample from each quadrat. In total, 55 samples were collected for subsequent analyses. To minimize the confounding effects of spatial heterogeneity, all selected patches were flat fields with consistent topography, soil type, and vegetation conditions.

### 2.3 Soil DNA extraction and amplicon sequencing

Total microbial DNA was extracted from 0.5g soils using the FastDNA^®^ SPIN Pin Kit for Soil (MP Biomedicals, CA, USA) according to the standardized protocol. The resulting DNA extracts were dissolved in DES buffer and subsequently quantified and qualified using a NanoDrop spectrophotometer (NanoDrop Technologies, Wilmington, Germany).

The V4-V5 regions of the bacterial 16S rRNA gene were amplified using the primers 515F (GTGCCAGCMGCCGCGG) and 907R (CCGTCAATTCMTTTRAGTTT) (Caporaso et al., 2011). For fungi, the ITS2 region of the ITS gene was amplified using primers ITS1F (5-CTTGGTCATTTA-GAGGAAGTAA-3′) and ITS2R (5-GCTGCTTCTTCATCGATGC-3′) (White et al., 1990; Zhang et al., 2016). The PCR amplification cycles were set as follows: an initial denaturation at 95°C for 2 min, followed by 25 cycles of 95°C for 30 s, 55°C for 30 s, and 72°C for 30 s, with a final extension at 72 °C for 10 min. The PCR products were then purified and quantified (Gao et al., 2021).

Purified PCR products were pooled in identical quantities and sequenced on the Illumina HiSeq2500 platform (Illumina Inc., San Diego, CA, USA). The raw paired-end reads were denoised and assembled using DADA2 v1.1.3 (Callahan et al., 2016). Chimeras were detected and removed with the USEARCH tool (Edgar, 2010). Raw reads were assigned to their respective samples according to barcodes using Lima, and forward and reverse sequences were merged using FLASH (Magoč and Salzberg, 2011). Taxonomic annotation for 16S rRNA sequences was performed based on the SILVA database (version 132) (Quast et al., 2012), while ITS sequences were annotated using the UNITE fungal database (Nilsson et al., 2019). Finally, Operational Taxonomic Units (OTUs) were clustered at a 97% similarity cutoff using UPARSE (Edgar, 2013, 2004). The resulting OTU table was used for all subsequent analyses.

### 2.4 Community diversity analysis

Alpha diversity was calculated in R to assess community richness and diversity within each sample. The metrics included Observed OTUs, the Chao1 richness estimator, the Shannon and Simpson diversity indices. Beta diversity was analyzed in Python to evaluate structural variations between microbial communities. A Bray-Curtis dissimilarity matrix was generated from the OTU abundance data (Bray and Curtis, 1957). Community structure was visualized using Principal Coordinates Analysis (PCoA) (Gower, 1966).

### 2.5 Community succession and ecological stage identification

To track the community's progression toward a stable state, we first quantified its convergence over the restoration chronosequence. A pseudocount was added to the OTU table, which was then subjected to a Centered Log-Ratio (CLR) transformation (Aitchison, 1982; Gloor et al., 2017). The Aitchison distance of each sample to the centroid of the non-degraded reference group was calculated to measure its successional distance.

To identify discrete ecological states within the continuous restoration process, we employed a consensus clustering approach, as the detection of clusters can be sensitive to the choice of algorithms. Two complementary unsupervised learning algorithms were applied to the Bray-Curtis dissimilarity matrix: (1) Hierarchical Clustering (using Ward's minimum variance linkage) and (2) K-Means Clustering (applied to PCoA coordinates).

The optimal number of clusters (*k*) was determined by evaluating the Silhouette Score across a range of potential values (from *k* = 2 to *k* = 6). The analysis revealed that the Silhouette Score reached its maximum at *k* = 2 for both bacterial and fungal communities, whereas higher *k* values (e.g., *k* = 3 or 4) led to a sharp decline in score, indicating reduced cluster cohesion. Consequently, *k* = 2 was selected as the statistically optimal solution.

Ecological stages were defined based on the consensus of these methods and their temporal distribution. A sample was assigned to a Core Stage only if it was consistently classified into the same group by both algorithms; samples with conflicting classifications were designated as Ambiguous.

The ecological identity of these two identified clusters was defined based on their distinct network topological structures and assembly mechanisms, rather than their chronological age. The first cluster was designated as the Chaos Stage. This definition is empirically grounded in its structurally fragmented topology, characterized by small average module size, sparse connectivity, and weaker topological constraints (lower Modularity Z-score relative to the Recovery stage). This reflects a state of weak organization typical of early succession or high-stress environments. The second cluster was designated as the Recovery Stage. This definition is based on its cohesive network architecture, characterized by high density, strong topological constraints, and the emergence of keystone taxa and competitive exclusions, indicating a shift toward a stable, deterministic, and self-regulating regime comparable to the non-degraded reference.

This structure-based definition strategy avoids the assumption of linear recovery over time and allows for the detection of nonlinear dynamics, such as structural reversions (secondary degradation) where the community network collapses into a chaotic state despite the passage of restoration time.

### 2.6 Biomarker identification and model validation

A Random Forest classifier (*n*_estimators_ = 100) (Breiman, 2001) was trained to identify the key OTUs (biomarkers) that best discriminate between the Chaos and Recovery stages. The relative importance of each OTU was quantified using the Gini impurity-based feature importance metric.

The predictive performance of these biomarkers was rigorously assessed using a stratified 5-fold cross-validation. The model's ability to classify samples was evaluated using the Area Under the Receiver Operating Characteristic Curve (AUC) (Fawcett, 2006) and a cumulative confusion matrix aggregated from all cross-validation folds.

### 2.7 Network construction and analysis

To reveal potential microbial interactions, co-occurrence networks were constructed independently for the Chaos and Recovery stages. A stringent filtering protocol was first applied to select core OTUs for each stage. An OTU was retained only if it was present in >50% of the stage's samples (prevalence) and had a mean relative abundance >0.01% (abundance). Pairwise Spearman's rank correlations were calculated among these core OTUs. A significant interaction (an edge in the network) was defined as a correlation with an absolute Spearman's ρ > 0.7 and an FDR-adjusted *P*-value <0.01 using the Benjamini-Hochberg procedure (Benjamini and Hochberg, 1995).

The topological properties of each network were analyzed to compare their structure. Modules of highly interconnected OTUs were identified using the Louvain community detection algorithm (Blondel et al., 2008). To identify keystone taxa, the within-module degree (*Z*_*i*_) and participation coefficient (*P*_*i*_) were calculated for each OTU (node) (Guimera and Nunes Amaral, 2005). Based on these metrics, nodes were classified into four ecological roles: peripherals (*Z*_*i*_ ≤ 2.5, *P*_*i*_ ≤ 0.62), connectors (*Z*_*i*_ ≤ 2.5, *P*_*i*_>0.62), module hubs (*Z*_*i*_>2.5, *P*_*i*_ ≤ 0.62), and network hubs (*Z*_*i*_>2.5, *P*_*i*_>0.62). The networks were visualized using a force-directed layout algorithm (Fruchterman and Reingold, 1991), where node size was proportional to its degree, node color indicated its module affiliation, and edge color and width represented the sign and strength of the correlation, respectively.

To confirm the deterministic nature of the identified stages and evaluate the non-randomness of the network topologies, we employed a Topological Null Model Analysis (Deng et al., 2012). Specifically, we generated random networks using the Erdös–Rényi model, preserving the identical number of nodes (*N*) and edges (*M*) as the empirical networks to establish a “baseline of randomness” (Zhang et al., 2024). We then calculated the Modularity Z-score to quantify the magnitude of deviation of the observed network structure from this stochastic baseline. The Z-score is calculated as:


$$Z=\frac{Q_{obs}-{\bar{Q}}_{rand}}{\sigma_{rand}}$$

where *Q*_*obs*_ is the observed modularity of the empirical networks, and ${\bar{Q}}_{rand}$ and σ_*rand*_ are the mean and standard deviation of the modularity derived from the null model ensemble (*n* = 100). A higher Z-score indicates a stronger constraint by deterministic rules rather than random accumulation of links.

To quantitatively assess the topological vulnerability of the Recovery Stage networks, a series of node-removal simulations were performed. Network robustness was quantified by tracking the relative size of the largest connected component (LCC) as nodes were progressively removed. Three distinct removal strategies were implemented: (1) Random Failure, where nodes were removed in random order (averaged over 50 simulations); (2) Degree Attack, where nodes were removed in descending order of their degree; and (3) Keystone Attack, where nodes were removed based on their ecological importance (Network Hubs > Module Hubs > Connectors > Peripherals), with degree used as a tie-breaker within each role.

### 2.8 Statistical analysis

All statistical analyses were performed in the R (v4.5.0) and Python (v3.12) environments. The primary R packages used included vegan, ggplot2, and FSA, while Python analyses relied on pandas, scikit-bio, scikit-learn, and matplotlib. To identify significant differences in alpha diversity metrics among groups, a non-parametric Kruskal-Wallis test was used, followed by a Dunn's post-hoc test with Benjamini-Hochberg (BH) correction. To test for significant group-level differences in beta diversity, we performed both a Permutational Multivariate Analysis of Variance (PERMANOVA) (Anderson, 2001) and an Analysis of Similarities (ANOSIM) (Clarke, 1993), each with 999 permutations, on the Bray-Curtis dissimilarity matrix. To determine the optimal number of clusters (k) for K-Means clustering, the k value that maximized the silhouette score was selected. A Random Forest classifier (*n*_estimators_ = 100) was trained to identify biomarkers (Breiman, 2001). The relative importance of each OTU was quantified using the Gini impurity-based feature importance metric. Model performance was rigorously assessed using a stratified 5-fold cross-validation, and its predictive ability was evaluated using the Area Under the Receiver Operating Characteristic Curve (AUC) (Fawcett, 2006) and a cumulative confusion matrix. Network edges were defined based on pairwise Spearman's rank correlations. A significant interaction (an edge) was defined as a correlation with an absolute Spearman's ρ > 0.7 and an FDR-adjusted *P*-value <0.01 using the Benjamini-Hochberg procedure (Benjamini and Hochberg, 1995). Modules of highly interconnected OTUs were identified using the Louvain community detection algorithm (Blondel et al., 2008).

## 3 Results

### 3.1 Divergent alpha diversity trajectories of bacterial and fungal communities

**Figure 1:**
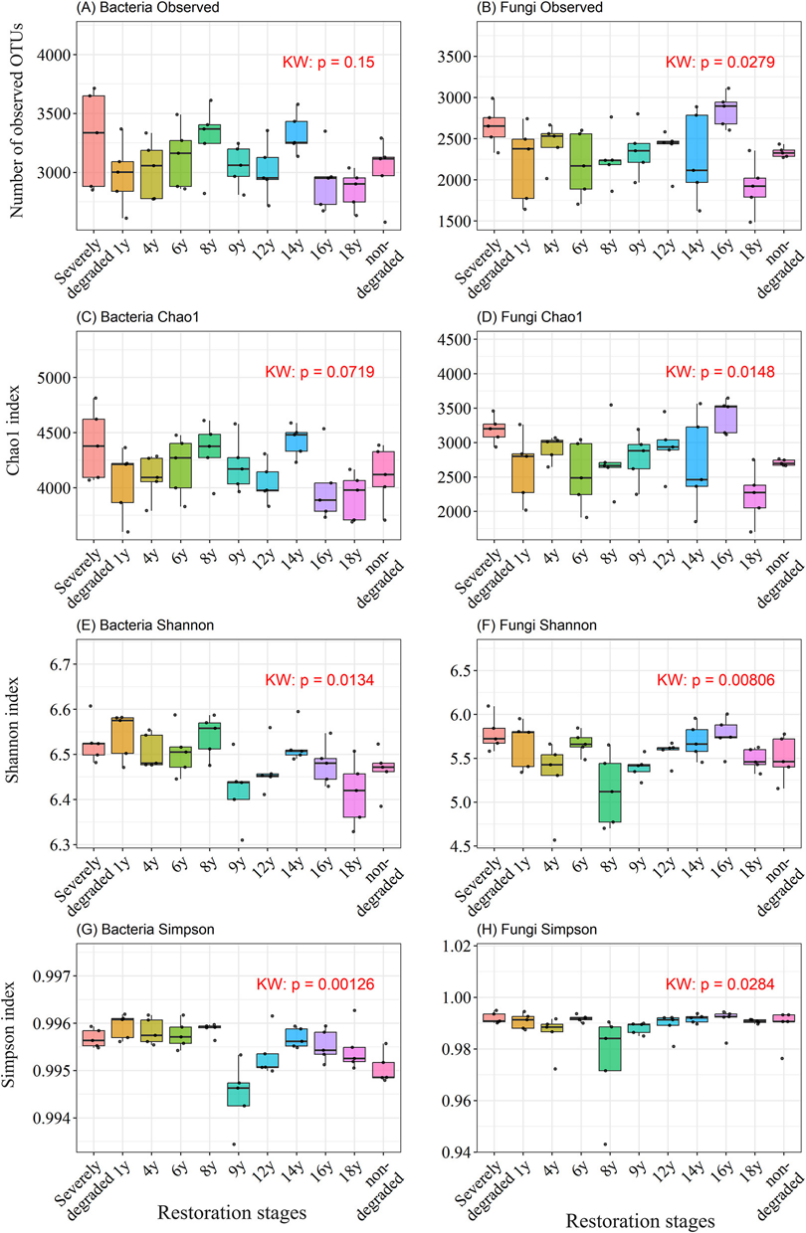
Alpha diversity of bacterial and fungal communities across the restoration chronosequence. The figure displays four alpha diversity metrics for bacterial **(A, C, E, G)** and fungal **(B, D, F, H)** communities. **(A, B)** Observed OTUs. **(C, D)** Chao1 richness estimator. **(E, F)** Shannon diversity index. **(G, H)** Simpson diversity index. Boxplots represent the distribution of values for each restoration stage, with the center line indicating the median. Individual points are overlaid as jittered dots. The Kruskal-Wallis (KW) test *p*-value for the overall comparison among all groups is shown in the upper right corner of each panel. Letters above the boxplots indicate significant differences based on a Dunn's *post-hoc* test (*p* < 0.05); groups that do not share a letter are significantly different.

The alpha diversity of microbial communities varied across the restoration chronosequence (Figure 1). The richness of the bacterial communities, as measured by Observed OTUs and the Chao1 index, did not show a statistically significant change across the restoration stages (Kruskal-Wallis, *p*>0.05) (Figures 1A, C). In contrast, bacterial diversity, measured by the Shannon and Simpson indices, exhibited a significant change (Kruskal-Wallis, *p* < 0.05) (Figures 1E, G). Conversely, both the richness and diversity of the fungal community showed significant dynamic changes (Kruskal-Wallis, *p* < 0.05) (Figures 1B, D, F, H). These results indicate that bacterial and fungal communities follow divergent successional trajectories in terms of their alpha diversity.

### 3.2 Community structure and taxonomic composition

**Figure 2:**
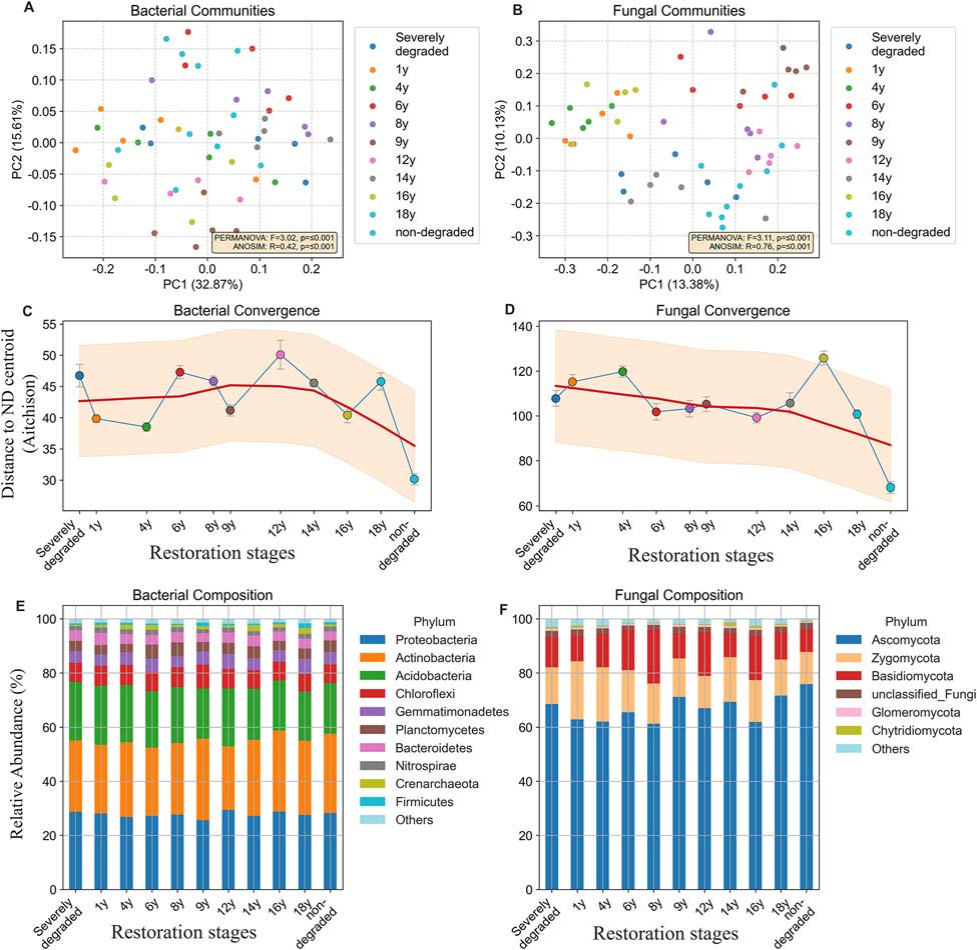
Beta diversity, successional convergence, and taxonomic composition of microbial communities. **(A, B)** Principal Coordinates Analysis (PCoA) of bacterial **(A)** and fungal **(B)** communities based on Bray-Curtis dissimilarity. Each point represents a sample, colored by its restoration stage. The percentage of variation explained by each principal coordinate is shown on the axes. Results from PERMANOVA and ANOSIM tests are displayed in the lower right. **(C, D)** Community convergence over time for bacteria **(C)** and fungi **(D)**, measured as the Aitchison distance of each sample to the centroid of the non-degraded reference group. Points represent the mean distance for each stage, and the red line indicates the LOWESS smoothing trend. **(E, F)** Average taxonomic composition at the phylum level for bacterial **(E)** and fungal **(F)** communities in each restoration stage. Taxa with low abundance are grouped into Others.

Principal Coordinates Analysis (PCoA) based on Bray-Curtis dissimilarity revealed a distinct successional pattern for both bacterial and fungal communities across the restoration stages (Figures 2A, B). The separation of communities over time was statistically significant for both kingdoms. For bacteria, the PERMANOVA test yielded an *F*-statistic of 3.02 (*p* = 0.001), and the ANOSIM test resulted in an *R*-statistic of 0.42 (*p* = 0.001). The fungal community showed an even stronger separation between stages, with a PERMANOVA *F*-statistic of 3.11 (*p* = 0.001) and an ANOSIM *R*-statistic of 0.76 (*p* = 0.001). The higher *R*-statistic for fungi indicates a more pronounced community structure differentiation between restoration stages compared to bacteria.

The analysis of community convergence, measured by the Aitchison distance to the non-degraded reference centroid, confirmed a directional succession for both communities (Figures 2C, D). The overall trend, indicated by the smoothing line, shows that the distance to the reference state generally decreases with increasing restoration time, suggesting a gradual convergence of both the bacterial and fungal communities toward the structure of the non-degraded ecosystem.

At the phylum level, the bacterial community composition remained remarkably stable throughout the entire restoration process (Figure 2E). The community was consistently dominated by Proteobacteria, Actinobacteria, and Acidobacteria. The relative abundances of these major phyla showed only minor fluctuations and no clear directional trend across the stages. For instance, the most abundant phylum, Proteobacteria, maintained a relative abundance that fluctuated between approximately 25% and 29% across all stages. In contrast, the fungal community displayed a dynamic and nonlinear successional pattern at the phylum level (Figure 2F). While Ascomycota was the dominant phylum in all stages, its relative abundance fluctuated over time. Other major phyla exhibited distinct successional trajectories. Zygomycota showed its highest relative abundance in the early stages of restoration (21.5% at 1 year) and generally decreased in the later stages. Basidiomycota peaked in the intermediate stages of succession, reaching 19.3% at 8 years and 16.4% at 16 years, before declining in the non-degraded stage. These dynamic shifts indicate a significant and complex compositional turnover within the fungal community as the ecosystem matures.

### 3.3 Identification of distinct ecological stages via unsupervised clustering

**Figure 3:**
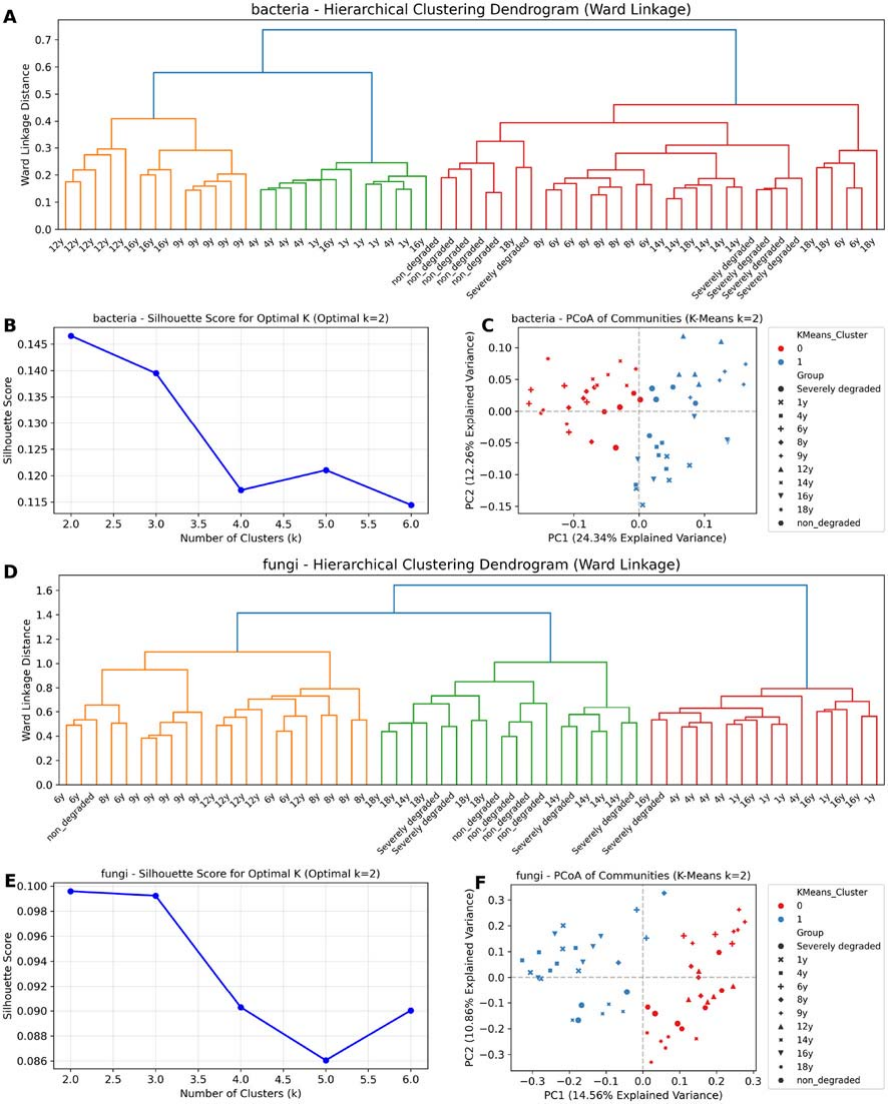
Unsupervised clustering of bacterial and fungal communities reveals two core ecological stages. **(A, D)** Hierarchical clustering dendrograms based on Ward's linkage of Bray-Curtis distances for bacterial **(A)** and fungal **(D)** communities. The two primary superclusters are indicated by different colors. **(B, E)** Silhouette score analysis used to determine the optimal number of clusters (*k*) for K-means clustering for bacteria **(B)** and fungi **(E)**. The peak score at *k* = 2 indicates that two clusters provide the most robust separation. **(C, F)** PCoA plots showing the distribution of bacterial **(C)** and fungal **(F)** communities, with each sample colored according to its assignment to one of the two optimal K-means clusters.

Unsupervised clustering analyses consistently partitioned both bacterial and fungal communities into two distinct ecological stages that corresponded to the restoration timeline (Figure 3).

For the bacterial communities, hierarchical clustering revealed a primary bifurcation that separated early-to-middle stage samples from those in the later, recovered stages (Figure 3A). The optimal number of clusters was determined to be two, as indicated by the peak silhouette score at *k* = 2 (Figure 3B). When mapped onto the PCoA ordination, these two clusters exhibited a clear separation along the first principal coordinate (PC1), with Cluster 0 corresponding to the early successional samples and Cluster 1 to the later stages (Figure 3C).

The fungal communities displayed an equally clear division. Both hierarchical clustering (Figure 3D) and silhouette score analysis (Figure 3E) supported the partitioning of the fungal data into two optimal clusters. These clusters also separated distinctly along PC1, with Cluster 1 representing the early stages and Cluster 0 representing the later stages of succession (Figure 3F).

Based on the consensus between both clustering methods, we defined two core ecological stages for subsequent analyses. This resulted in the delineation of an early Chaos Stage (*n* = 25 for bacteria, *n* = 16 for fungi) and a later Recovery Stage (*n* = 25 for bacteria, *n* = 29 for fungi). Samples not consistently classified by both methods were designated Ambiguous and excluded from subsequent stage-based analyses.

### 3.4 Distinct and predictable microbial signatures of ecological stages

**Figure 4:**
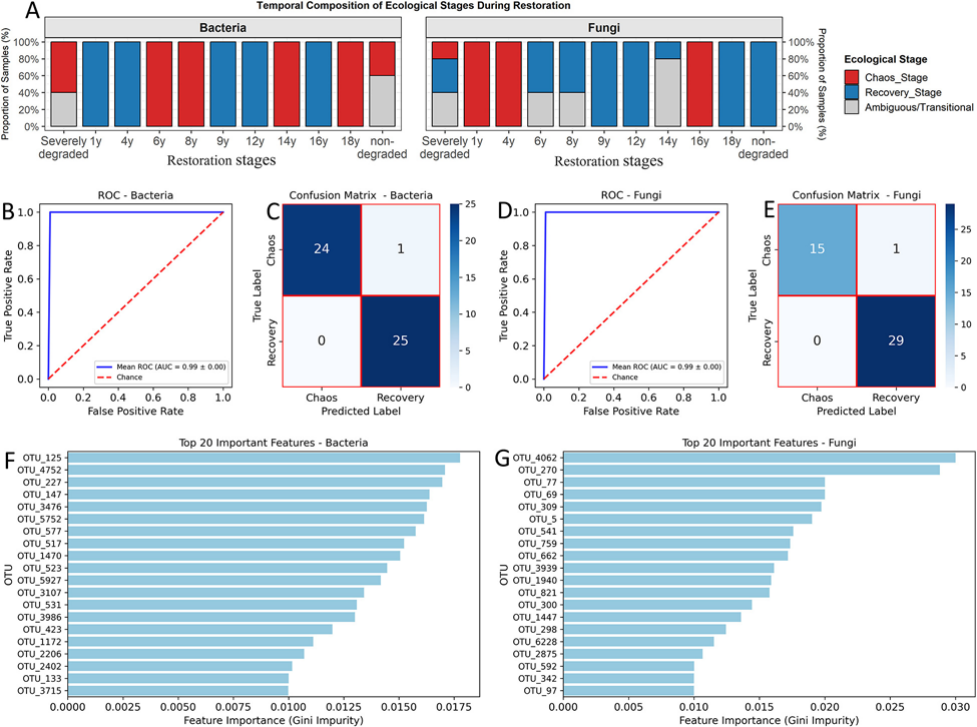
Validation of ecological stages and identification of biomarker OTUs using a Random Forest model. **(A)** The stacked bar charts illustrate the proportional distribution of three ecological stages (Chaos Stage, Recovery Stage, and Ambiguous) for both bacterial (left) and fungal (right) communities. **(B, D)** Receiver Operating Characteristic (ROC) curves from a 5-fold cross-validation for the classification of bacterial **(B)** and fungal **(D)** communities into Chaos and Recovery stages. The mean Area Under the Curve (AUC) and its standard deviation are shown. **(C, E)** Aggregated confusion matrices showing the classification performance for bacteria **(C)** and fungi **(E)** across all cross-validation folds. **(F, G)** The top 20 most important biomarker OTUs for distinguishing the two stages in bacterial **(F)** and fungal **(G)** communities, ranked by their feature importance (Gini impurity).

To visualize the temporal distribution of the ecological stages identified by clustering, we plotted the proportion of samples from each time point assigned to the Chaos Stage, Recovery Stage, or Ambiguous category (Figure 4A). The results revealed complex, nonlinear successional dynamics that were highly divergent between bacteria and fungi. For the bacterial communities, the assignment to ecological stages did not follow a simple linear progression. The Recovery Stage (blue) was predominant in the early (1y, 4y) and several middle-to-late stages (9y, 12y, 16y). However, the Chaos Stage (red) was dominant not only in the initial Severely degraded state but also recurred in intermediate stages (6y, 8y) and, notably, in the final restoration stages (14y, 18y) and the non-degraded reference state. The fungal communities exhibited a different, though also nonlinear, pattern. The Chaos Stage (red) dominated the initial Severely degraded state and the first 4 years of recovery (1y, 4y). A clear shift occurred in the middle-to-late stages (6y to 14y), which were strongly dominated by the Recovery Stage (blue). However, a distinct structural reversion to the Chaos Stage was observed at the 16y mark, before the community returned to the Recovery Stage at 18y and in the non-degraded reference state.

For the bacterial communities, the classification accuracy in distinguishing between the two stages was near-perfect. The mean Area Under the Receiver Operating Characteristic Curve (AUC) was 0.99 ± 0.00 (Figure 4B). The aggregated confusion matrix from all cross-validation folds confirmed this, showing that only one of the 50 core samples was misclassified (Figure 4C). This strong separation was driven by a specific set of biomarker OTUs, with OTU_125 and OTU_4752 identified as the most influential predictors (Figure 4F). The fungal communities showed a similarly high degree of predictability. The model achieved a mean AUC of 0.99 ± 0.00 (Figure 4D), and the confusion matrix revealed only one misclassification out of 45 core samples (Figure 4E). A distinct set of fungal OTUs served as the primary biomarkers, with OTU_4062, OTU_270, and OTU_69 being the most important features for differentiating the Chaos and Recovery stages (Figure 4G). These results validate that the defined ecological stages are characterized by unique and highly predictable microbial signatures, and they identify the specific OTUs that serve as the strongest biomarkers for each successional phase.

### 3.5 Shifts in microbial co-occurrence network structure

**Figure 5:**
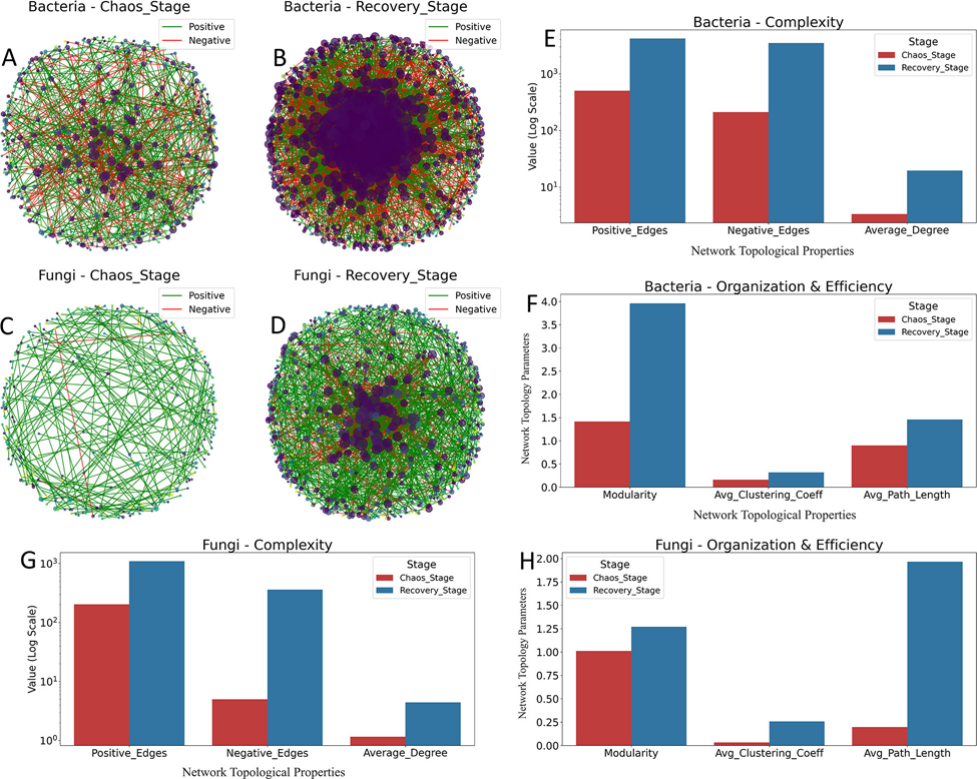
Microbial co-occurrence networks in Chaos and Recovery stages. **(A–D)** Network visualizations showing co-occurrence patterns for bacteria in the Chaos stage **(A)** and Recovery stage **(B)**, and for fungi in the Chaos stage **(C)** and Recovery stage **(D)**. Nodes represent OTUs, colored by module affiliation. Green edges indicate positive correlations (Spearman's ρ>0.7), and red edges indicate negative correlations (ρ <−0.7). Node size is proportional to its degree. **(E–H)** Comparison of global network topological properties for bacterial complexity **(E)**, bacterial organization **(F)**, fungal complexity **(G)**, and fungal organization **(H)** between the Chaos and Recovery stages.

The transition from the Chaos Stage to the Recovery Stage was characterized by a profound increase in network complexity (Figure 5).

Visual analysis revealed a dramatic increase in network complexity during succession. The bacterial and fungal networks of the Chaos Stage were relatively sparse, whereas the Recovery Stage networks were significantly larger and more densely connected (Figures 5A–D). This was quantitatively supported by a large increase in the number of both positive and negative edges, as well as a higher average degree in the Recovery Stage for both kingdoms (Figures 5E, G). Crucially, the composition of these interactions shifted toward competitive dominance. As shown in Table 1, while absolute numbers increased for both edge types, the proportion of negative edges rose significantly from 29.5% (211/715) to 45.6% (3,502/7,688) in bacterial networks. This shift was even more pronounced in fungal networks, where the negative edge proportion increased ten-fold from 2.4% (5/208) to 24.8% (360/1,449).

**Table 1:** Positive and negative edges in co-occurrence networks between ecological stages.

**Kingdom**	**Stage**	**Positive edges**	**Negative edges**	**Total edges**	**Proportion**
Bacteria	Chaos stage	504	211	715	29.5%
	Recovery stage	4,186	3,502	7,688	45.6%
Fungi	Chaos stage	203	5	208	2.4%
	Recovery stage	1,089	360	1,449	24.8%

The organizational properties of the networks also shifted significantly. For both bacteria and fungi, the Recovery Stage networks exhibited a higher average clustering coefficient and a longer average path length (Figures 5F, H). The increased clustering coefficient suggests a greater degree of local connectivity and guild formation. The longer average path length reflects the profound increase in the overall size and complexity of the networks, indicating that interactions span across a more expansive topological space. Concurrently, modularity increased in the Recovery Stage, suggesting a transition from a community of loosely associated guilds to a more compartmentalized and specialized system. This increased modularity is a hallmark of mature ecosystems, as it can enhance stability by containing perturbations within specific functional guilds.

To validate the non-randomness of these topological structures, we further compared the observed modularity against null models. While networks in both stages exhibited modular structures significantly different from random graphs (*Z*-score>2), the strength of these topological constraints varied dramatically (Table 2). The Recovery Stage displayed exceptionally high structural robustness, with Modularity Z-scores reaching 39.69 for bacteria and 39.75 for fungi. In contrast, the Chaos Stage showed significantly weaker structural constraints, with much lower Z-scores (16.10 for bacteria and 5.15 for fungi), confirming the relatively loose and fragmented organization of communities in this early phase.

**Table 2:** Topological properties and null model analysis between Chaotic and Recovery stages.

**Kingdom**	**Stage**	**Nodes**	**Edges**	**No. of modules**	Avg. module size^*^	**Modularity Z-score**
Bacteria	Chaotic stage	426	715	119	3.58	16.10
	Recovery stage	785	7,688	145	5.41	39.69
Fungi	Chaotic stage	359	208	165	2.18	5.15
	Recovery stage	657	1,449	161	4.08	39.75

*Avg. Module Size, Nodes/No. of Modules. Smaller size indicates higher fragmentation.

### 3.6 Identification, characterization, and structural importance of keystone taxa

**Figure 6:**
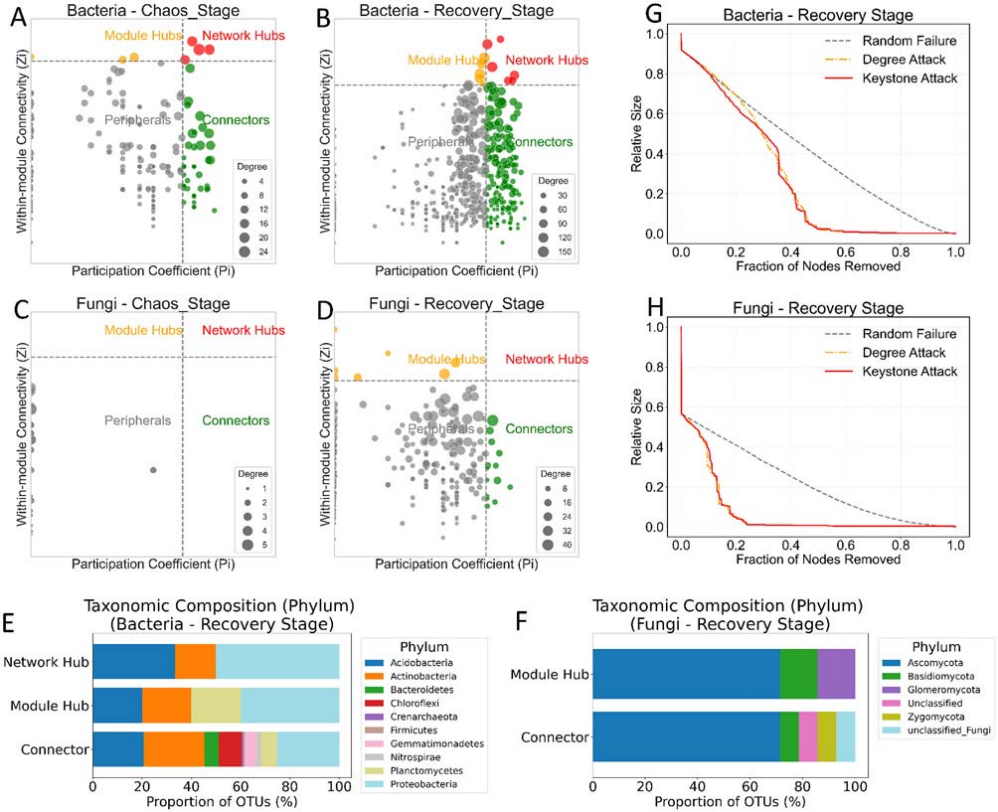
Identification of keystone taxa, their taxonomic composition, and their impact on network robustness. **(A–D)** *Z*_*i*_-*P*_*i*_ plots showing the ecological roles of each node (OTU) in the bacterial Chaos stage **(A)**, bacterial Recovery stage **(B)**, fungal Chaos stage **(C)**, and fungal Recovery stage **(D)**. Nodes are classified based on their within-module connectivity (*Z*_*i*_) and among-module connectivity (*P*_*i*_) as Peripherals (*Z*_*i*_ ≤ 2.5, *P*_*i*_ ≤ 0.62), Connectors (*Z*_*i*_ ≤ 2.5, *P*_*i*_>0.62), Module Hubs (*Z*_*i*_>2.5, *P*_*i*_ ≤ 0.62), or Network Hubs (*Z*_*i*_>2.5, *P*_*i*_>0.62). Node size is proportional to its degree. **(E, F)** Phylum-level taxonomic composition of the keystone taxa (Connectors, Module Hubs, and Network Hubs) identified in the Recovery Stage network, shown for bacteria **(E)** and fungi **(F)**. Each horizontal bar represents 100% of the OTUs identified within that specific ecological role. **(G, H)** Network robustness analysis of the Recovery Stage networks for bacteria **(G)** and fungi **(H)**. The plots show the decline in the relative size of the largest connected component (LCC, Y-axis) as a fraction of nodes is removed (X-axis). Three removal strategies are compared: targeted removal of keystone taxa (Keystone Attack, red solid line), targeted removal by highest degree (Degree Attack, orange dash-dot line), and random removal (Random Failure, gray dashed line).

To elucidate the mechanisms of network reassembly, we identified the ecological roles of all nodes (OTUs) in both the Chaos and Recovery Stage networks, characterized their taxonomic identity, and tested their structural importance (Figure 6).The ecological roles of nodes, as determined by *Z*_*i*_-*P*_*i*_ analysis, shifted dramatically between the two stages (Figures 6A–D). For bacteria, the Chaos Stage network already possessed some Connectors, Module Hubs, and Network Hubs (Figure 6A). In the Recovery Stage, the number of nodes classified as Connectors and Module Hubs increased (Figure 6B). A more profound shift was observed in the fungal communities. The Chaos Stage network was composed almost entirely of Peripherals, with virtually no keystone taxa identified (Figure 6C). In stark contrast, the Recovery Stage network showed a clear emergence of numerous Connectors and Module Hubs (Figure 6D).

To identify the key taxa associated with different network topological roles, we analyzed the phylum-level taxonomic composition of OTUs classified as network hubs, module hubs, or connectors for both bacteria (Figure 6E) and fungi (Figure 6F). In the bacterial network (Figure 6E), distinct taxonomic profiles were observed for each role. Connectors exhibited the highest taxonomic diversity, with OTUs distributed across 10 major phyla. In contrast, bacterial hub nodes were taxonomically less diverse. Network hubs were predominantly composed of OTUs from Acidobacteria and Proteobacteria. Module hubs were also dominated by Proteobacteria, but included substantial fractions of Acidobacteria, Actinobacteria, and Planctomycetes. A similar trend was observed in the fungal community network (Figure 6F), which compared module hubs and connectors. Fungal module hubs were overwhelmingly dominated by Ascomycota, followed by Basidiomycota and Glomeromycota. In parallel with the bacterial findings, fungal connectors, while also dominated by Ascomycota, displayed greater taxonomic diversity. They incorporated several phyla that were absent in the hubs. Collectively, these results indicate that while the specific dominant phyla differ between bacteria and fungi, the network connectors in both kingdoms are composed of a more taxonomically diverse range of taxa compared to the more taxonomically-conserved hub nodes.

Finally, the structural importance of these keystone taxa was validated using network robustness analysis (Figures 6G, H). For the bacterial network (Figure 6G), the structure was highly robust to random failure, as indicated by a slow, linear decline in relative network size. In sharp contrast, the network was highly fragile when subjected to targeted attacks. The Degree Attack and Keystone Attack curves were nearly identical, indicating that the most connected nodes were the keystone species in this community. The bacterial network began to collapse after approximately 38% of keystone nodes were removed, and it was almost completely fragmented after 50% removal. The fungal network (Figure 6H) exhibited a similar pattern but showed even greater fragility. While also resilient to random failure, the fungal network was extremely sensitive to targeted attacks. The removal of just 10% of its keystone nodes caused the relative network size to plummet to 0.4, and the network structure collapsed almost entirely upon the removal of only 20% of these nodes. These results demonstrate that while both microbial networks are resilient to stochastic species loss, their integrity is critically dependent on a small fraction of highly connected keystone species. The fungal network, in particular, displayed extreme vulnerability to the loss of these key taxa.

## 4 Discussion

Our study provides a multi-faceted view of microbial community succession during the ecological restoration of a degraded alpine grassland ecosystem. By integrating analyses of diversity, community structure, and network interactions, we reveal a clear, predictable trajectory from a simple, disorganized state to a complex, integrated community. The results strongly support a two-stage model of succession—an initial Chaos Stage governed by stochastic processes and a subsequent Recovery Stage characterized by the emergence of deterministic interactions and keystone taxa.

### 4.1 Rationale for clustering: moving beyond linear chronosequence

Community succession during restoration often exhibits nonlinear characteristics, particularly in the dynamics of microbial communities. Scheffer et al. (2001) proposed the theory of tipping points in ecosystems, emphasizing that during restoration, systems may undergo abrupt shifts rather than simple linear progression. This theory provides a framework for understanding nonlinear community recovery. Further research indicates that microbial communities often display complex resistance and resilience during recovery, regulated by soil properties and disturbance history, and exhibit significant hysteresis effects. This means microbial communities may stagnate or experience secondary degradation during certain recovery phases (Griffiths and Philippot, 2013).

The nonlinear response of microbial communities is more pronounced in unique habitats. For instance, in restoration studies of alpine grasslands, similar nonlinear behaviors have been confirmed: microbial communities in alpine ecosystems frequently show phased fluctuations and divergent recovery paths, where localized areas may remain in specific stable states due to unbroken environmental thresholds, or switch nonlinearly between different states (Wu et al., 2024; Fujita et al., 2025). Consequently, while traditional time-series analysis methods, such as linear chronosequence, can reveal some basic trends in restoration, they may fail to accurately capture the internal nonlinear dynamics of the community.

To address this, cluster analysis, as an unsupervised learning method, can automatically identify discrete ecological stages within microbial communities or ecosystems based on data similarity, revealing abrupt shifts and nonlinear changes during community assembly. Clustering methods have been widely applied in ecology to identify nonlinear dynamics in ecological restoration, especially in complex community succession, where they can reveal phased transitions. For example, Koren et al. (2013), in their study of human microbial communities, noted that while most microbial communities show smooth gradients in taxon abundance, some specific communities exhibit bimodal or multimodal distributions. This suggests that such communities may possess discrete ecological states, providing a theoretical basis for identifying community stages through clustering. Shi et al. (2022) further elaborated on this, stressing that the identification of clustering results is strongly influenced by the chosen analytical methods. Through systematic comparison, they found that no universally optimal clustering method exists for all microbial data, necessitating method selection and validation based on data-specific characteristics.

Therefore, to ensure our conclusions are robust and reliable, we adopted a multi-step cross-validation approach to validate the effectiveness of the cluster analysis. First, we selected two unsupervised methods, hierarchical clustering and K-means clustering, to analyze the restoration process of the alpine grassland. Through these clustering methods, we automatically identified two significant ecological stages in the restoration process: the Chaotic Stage and the Restoration Stage.

To further validate the reliability of these clustering results, we utilized a Random Forest (RF) model as a supervised learning method. The RF model excels in classifying and selecting features in high-dimensional microbial data, effectively distinguishing noise from true community differences, making it an ideal tool for verifying the authenticity of clustering results. By using the clustering results as labels, we classified the microbial community data. The model's classification accuracy was near-perfect (AUC > 0.99), and it successfully identified highly predictive biomarker OTUs associated with the Chaotic Stage and Restoration Stage. This outcome aligns with the research logic of Wu et al. (2011) in verifying the authenticity of human gut microbiome groupings. By using 16S rDNA sequencing combined with PAM clustering, Wu et al. (2011) confirmed that enterotypes are not random statistical groupings but ecologically genuine states closely linked to long-term diet, distinguishable by characteristic microbial taxa. Similarly, our study, through model validation and biomarker OTU identification, demonstrates that the Chaotic Stage and Restoration Stage identified by clustering are not merely statistical groupings but are ecologically real, predictable states closely correlated with changes in community structure.

Through cluster analysis, our study clearly delineates two core ecological stages during the restoration, aligning with our understanding that ecological recovery is not a single, smooth process, but rather a complex one fraught with nonlinear changes. When analyzing the same dataset, Du et al. (2025) emphasized the nonlinear features of the restoration process, particularly noting the secondary degradation phenomenon and fluctuations occurring around years 8–9. The results of our cluster analysis further confirm and meticulously characterize this. For example, the fungal community at year 16 clearly reverted to the Chaotic Stage structure. Furthermore, the Chaotic Stage for the bacterial community recurred even in later stages (e.g., years 14, 18) and in the non-degraded grasslands. This indicates that restoration is not a steady progression toward the non-degraded state, but rather a process of dynamic fluctuation between two distinct states: Chaotic and Restoration. This phenomenon represents a genuine state shift in community structure between two stable states, reflecting the highly nonlinear characteristics of the restoration process.

By combining cluster analysis with Random Forest validation, we not only successfully identified key ecological stages in the alpine grassland restoration but also effectively verified the nonlinear characteristics and state transitions of these stages. The use of this methodology allows us to transcend the limitations of linear chronosequence, offering a more refined, data-driven approach to uncovering the complex dynamics in ecological restoration. Through the identification of ecological stage shifts and key microbial biomarkers, we provide powerful tools and guidance for future restoration management.

### 4.2 Decoupled temporal dynamics and kingdom-specific assembly strategies

The temporal composition of ecological stages reveals that bacterial and fungal communities follow distinct, nonlinear trajectories during restoration, reflecting fundamental differences in their life-history strategies and functional roles.

Bacterial communities exhibited the “fast-in, fast-out” dynamics characteristic of r-strategists (Fierer et al., 2007), where the immediate dominance of the Recovery Stage in early years (1y, 4y) reflects their role as rapid-responding pioneers establishing tightly coupled networks for early biogeochemical cycling (Morriën et al., 2017; Bastida et al., 2016). However, this structural coupling proved highly volatile, as evidenced by the frequent recurrence of the Chaos stage (6y, 8y, 14y, 18y), suggesting that bacterial associations are transient responses to environmental fluctuations rather than permanent structures (De Vries et al., 2012, 2018). Crucially, the reversion to a chaotic or ambiguous state in the non-degraded reference supports a “Relaxation Hypothesis,” implying that in resource-rich, healthy environments, bacteria alleviate metabolically expensive network constraints and relax' into a functionally redundant state (Zhou and Ning, 2017; Dini-Andreote et al., 2015; Costello et al., 2012).

In contrast to bacteria, fungal communities demonstrated a “lagged but persistent” trajectory aligning with K-selected strategies (Fierer et al., 2007; Six et al., 2006), where the initial dominance of the Chaos Stage (years 1–4) reflects the physiological time constraints required for extensive hyphal network formation (Rousk and Bååth, 2011; Harris, 2009). However, once established, the Recovery Stage exhibited remarkable structural inertia that persisted into the non-degraded reference state, suggesting that complex fungal connectivity serves as the permanent “skeletal infrastructure” of healthy soil rather than a transient restoration mechanism (De Vries et al., 2012; Banerjee et al., 2019; Morriën et al., 2017). The singular reversion to the Chaos Stage at year 16 likely represents a punctuated successional turnover or temporary disturbance, underscoring that even stable, complex networks remain subject to nonlinear ecological dynamics (Dini-Andreote et al., 2015).

### 4.3 Network analysis reveals the mechanisms of ecological maturation

The transition from chaos to recovery is underpinned by profound shifts in network topology and interaction patterns. The co-occurrence network analysis reveals that the Recovery Stage is marked by a dramatic increase in network size, density and connectivity. These metrics indicate ecological maturation, suggesting a greater number of occupied niches and a more stable food web that enhances ecosystem resilience (Morriën et al., 2017; Wagg et al., 2019).

Crucially, this topological maturation is functionally defined by the disproportionate emergence of competitive interactions. While the absolute number of edges increased overall, the proportion of negative edges (relative to total associations) rose significantly during the transition from the Chaos to the Recovery Stage. Specifically, the proportion of negative edges increased from 29.5% to 45.6% in bacterial networks and, most strikingly, from 2.4% to 24.8% in fungal networks. This quantitative shift confirms that the community structure is increasingly governed by deterministic processes, such as competitive exclusion and niche partitioning (Faust et al., 2012; Dini-Andreote et al., 2015). This explicitly validates the Stress Gradient Hypothesis (Bertness and Callaway, 1994; He et al., 2013), consistent with findings by Du et al. (2025), where facilitation dominates in high-stress (Chaos) environments, whereas competition shapes the community in low-stress (Recovery) environments.

It is worth noting that while the Recovery Stage networks were denser and more clustered, they also exhibited a longer average path length. This seemingly counterintuitive result reflects the emergence of a single, expansive, and integrated network. Unlike the Chaos Stage, where short path lengths are artifacts of a fragmented structure composed of small, disconnected components, the longer path length in the Recovery Stage signifies a complex, cohesive topology where traversing the network naturally requires more steps (Banerjee et al., 2019). Similarly, keystone taxa and generalists play a pivotal role in bridging these extensive network structures to maintain stability (Herren and McMahon, 2018). Collectively, these network properties provide a mechanistic explanation for the predictable, two-stage succession observed in this restoration system.

### 4.4 Network vulnerability and the critical role of key nodes

Ecological restoration is a complex process involving the fundamental reorganization of community composition and species interaction networks. This study reveals that during alpine grassland restoration, microbial networks transition from a chaotic phase dominated by Peripherals to a highly structured phase led by Module Hubs and Connectors. This transition from a disorganized to a structured state not only signifies community functional maturation but also reshapes the ecosystem's stability and vulnerability.

In the severely stressed Chaotic phase, we observed that both fungal and bacterial networks lacked a clear core structure, a finding consistent with the general principle that extreme stress erodes microbial network structure (Banerjee et al., 2016, 2019; De Vries et al., 2018; Gao et al., 2022; Hernandez et al., 2021; Li et al., 2023). For instance, studies by De Vries et al. (2018) and Gao et al. (2022) have shown that stresses like drought destabilize microbial networks, leading to disordered connectivity and the loss of core nodes. Therefore, the peripheral-dominated topology observed in our chaotic phase further corroborates the pervasive disruptive effects of extreme environmental stress on the core structure of microbial networks.

As the community entered the recovery phase, however, a significant emergence of Module Hubs and Connectors signaled network reconstruction (Hou et al., 2025). We employed the topological methods of Guimera and Nunes Amaral (2005) and Deng et al. (2012) to dissect the ecological roles of these key nodes. We found that Hubs were taxonomically highly conserved (e.g., concentrated in Ascomycota), consistent with the concept of phylogenetic conservatism in core taxa (Philippot et al., 2010; Martiny et al., 2006). These specialist hubs form the core functional cornerstone of the community (Coyte et al., 2015). Conversely, Connectors exhibited high phylogenetic diversity, fitting their role as ecological generalists (Berry and Widder, 2014). They function as bridges connecting different functional modules (Olesen et al., 2007), thereby enhancing the entire network's connectivity.

This transition from chaos to structure represents a process of functional specialization. It relies on Hubs to stabilize module functions and on Connectors to integrate the entire network. However, this highly specialized structure, while conferring efficiency, also introduces an efficiency-vulnerability trade-off (Thébault and Fontaine, 2010; Dáttilo et al., 2016; Domínguez-García and Kéfi, 2024). Our robustness analysis explicitly confirmed this: both bacterial and fungal networks showed resilience to random species loss (Random Failure) but were highly vulnerable to the deliberate removal of core taxa (Keystone Attack). This phenomenon, where the loss of key nodes triggers network collapse, aligns perfectly with the classic study of scale-free networks by Albert et al. (2000) and findings in macro-ecological food webs by Dunne et al. (2002) and Hervías-Parejo et al. (2024), all confirming that network integrity is highly dependent on its key nodes.

The most significant finding of this study is the quantification of the stark difference in vulnerability between bacterial and fungal networks. Our simulations revealed that the fungal network exhibited extreme fragility—removing just 10% of key nodes caused the network size to plummet to 40%, and removing 20% led to near-total collapse. In contrast, the bacterial network, while also dependent on key nodes, demonstrated relatively stronger resilience. This finding provides a crucial mechanistic explanation and independent quantitative evidence for the study by Du et al. (2025). Analyzing the same dataset as this study, Du et al. (2025) identified from the perspective of community compositional resistance, resilience, and node constancy that bacterial community stability is significantly higher than fungal stability during alpine grassland restoration. Our study confirms and deepens this conclusion from the entirely new dimension of network topology and robustness. We not only explain why the phenomenon observed by Du et al. (2025) occurs (i.e., the bacterial network topology is more resilient) but also quantify the fungal network's collapse threshold (10%–20% key node loss), providing an operational standard for restoration early warning.

Notably, the bacteria-is-more-stable paradigm, which this study and Du et al. (2025)'s study jointly identified in this alpine grassland restoration system, stands in sharp contrast to recent findings in other ecosystems. For example, Li et al. (2024) found in their study of subtropical forests that neither warming nor nitrogen addition changed fungal network complexity and stability, while bacterial network stability was significantly affected, indicating that the fungal network exhibited higher intrinsic stability than the bacterial network under disturbance. This discrepancy strongly suggests that the relative stability of microbial networks is not a universal rule but is highly context-dependent.

## 5 Conclusion

In conclusion, this study elucidates the non-linear dynamics of microbial succession in alpine grassland restoration. We identified a critical transition from Chaos Stage to Recovery Stage, highlighting that successful restoration is driven by the progressive re-establishment of complex network interactions rather than the mere accumulation of species diversity. These findings offer a new theoretical framework for evaluating restoration success, suggesting that long-term monitoring should prioritize network stability and the presence of keystone taxa alongside traditional diversity metrics.

We identified divergent successional trajectories for bacteria and fungi, with fungi showing more dynamic changes in both diversity and composition. Critically, we validated that the Chaos and Recovery stages are not arbitrary groupings but represent distinct and highly predictable ecological regimes, each defined by a unique set of microbial biomarkers. The maturation of the ecosystem was marked by a profound increase in network complexity and the appearance of influential keystone species. However, this introduced a critical vulnerability trade-off, particularly within the fungal community, whose network stability became extremely dependent on these key nodes. This topological fragility provides a core mechanistic explanation for the lower stability observed in fungi compared to bacteria during restoration.

While this study provides a robust framework based on co-occurrence, future research incorporating multi-omics approaches (e.g., metatranscriptomics and metabolomics) is needed to validate the functional interactions implied by our network analysis. Furthermore, integrating these dynamic microbial network properties into ecosystem models will be crucial for improving predictions of biogeochemical cycling and for developing more effective strategies for managing and monitoring ecosystem restoration.

## Data Availability

Raw sequencing data presented in this study have been deposited in the NCBI Sequence Read Archive (SRA) repository under accession numbers PRJNA1199960 and PRJNA1199985.
